# Triglyceride-glucose index and related parameters in the diagnosis and predictive efficacy for older adults with NAFLD/MASLD: a prospective cohort study

**DOI:** 10.3389/fmed.2025.1626672

**Published:** 2025-08-26

**Authors:** Yuwei Chen, Xinjun Lin, Yanqiu Chen, Bingcai Wang, Chaoxiang Xu, Yaoguo Wang

**Affiliations:** 1 Department of Cardiology, The Second Affiliated Hospital of Fujian Medical University, Quanzhou, Fujian, China; 2 Department of Ultrasound Medicine, The Second Affiliated Hospital of Fujian Medical University, Quanzhou, Fujian, China

**Keywords:** triglyceride-glucose index, non-alcoholic fatty liver disease, triglyceride-glucose waistline, triglyceride-glucose BMI, triglyceride-glucose waist-to-height ratio, Fujian Province, China

## Abstract

**Background:**

Non-alcoholic fatty liver disease (NAFLD) affects over 25% of the global population and is associated with numerous comorbidities. Several indices, including the triglyceride-glucose (TyG) index, TyG-waist circumference (TyG-WC), TyG-body mass index (TyG-BMI), and TyG-waist-height ratio (TyG-WHtR), have been strongly associated with NAFLD. However, research on the stratification of hepatic steatosis severity in NAFLD, particularly in older adults, remains limited. This study aimed to investigate the relationship between TyG index and NAFLD in elderly individuals.

**Methods:**

This study analyzed physical examination data from 3,954 individuals aged 60 years or older, collected during a 2-year follow-up period at Nan’an General Hospital, Nanqiao Branch. Binary logistic regression and receiver operating characteristic curve analyses were employed to examine the relationship between the TyG index and NAFLD and its various pathological stages. Additionally, the change in the TyG index during follow-up was calculated to assess its predictive significance for NAFLD progression.

**Results:**

The TyG, TyG-WC, TyG-BMI, and TyG-WHtR quartiles were grouped for analysis in patients with NAFLD, revealing significant differences in hepatic steatosis severity between the groups (*p* < 0.001). All four parameters were significant predictors of hepatic steatosis severity (*p* < 0.001). After further adjusting for age, sex, marital status, alcohol consumption, smoking status, exercise, hypertension, diabetes, coronary heart disease, uric acid, HDL, ALT, and AST, the odds ratios (ORs) for the fourth quartile were 4.36 (3.39–5.61), 19.77 (14.92–26.22), 28.23 (20.81–28.28), and 14.56 (11.05–19.19), with all *p* values <0.001. The areas under the curve values for the four parameters for predicting NAFLD status were 0.744, 0.811, 0.840, and 0.813, respectively. Furthermore, an increase in the TyG index was identified as a risk factor for disease progression, while a decrease was associated with improvement in NAFLD severity.

**Conclusion:**

The TyG index and its related parameters serve as reliable, non-invasive indicators for the diagnosis and progression of NAFLD in elderly populations. Monitoring changes in these indices can improve early detection and management of NAFLD.

## 1 Introduction

Non-alcoholic fatty liver disease (NAFLD) is a significant global public health concern affecting over 25% of the world’s population (1). Histologically, NAFLD is classified into non-alcoholic fatty liver and non-alcoholic steatohepatitis (NASH) (2). NAFLD not only contributes to the development of type 2 diabetes and cardiovascular disease but may also progress to cirrhosis and hepatocellular carcinoma as the disease advances (3). Therefore, early detection and accurate prediction of NAFLD onset and progression are critical for reducing its health burden (4). Notably, recently, major liver societies such as the American Association for the Study of Liver Diseases (AASLD) and the European Association for the Study of the Liver (EASL) have approved the naming of NAFLD as metabolic dysfunction associated fatty liver disease (MASLD) (5, 6). However, in this study, since our initial diagnosis and data collection were performed before the widespread adoption of MASLD criteria. To ensure that the previous terminology is clearly familiar to the reader, we have retained the term NAFLD throughout the text, while acknowledging the newer terminology and its clinical significance.

The triglyceride-glucose (TyG) index has been demonstrated to be a valuable marker for predicting and aiding in the diagnosis of metabolic-associated fatty liver disease (7). It is a reliable predictor of cardiovascular and all-cause mortality (8). As research advances, the TyG index is increasingly recognized for its simplicity and accessibility (9), and it has emerged as one of the most accurate predictors of NAFLD (10). Additionally, TyG-related indicators, such as TyG-waist circumference (TyG-WC) (11), TyG-body mass index (TyG-BMI) (12), and TyG-waist-height ratio (TyG-WHtR) (13), are strongly correlated with NAFLD and considered valuable for assessing and diagnosing various NAFLD stages (14). The gold standard for diagnosing and assessing NAFLD is costly, time-consuming, and invasive, limiting its practicality for widespread screening (15).

With the rapid aging of the global population, the proportion of individuals aged 60 years and older is increasing at an unprecedented rate. According to the World Health Organization, the number of people aged 60 and above is expected to rise from 1 billion in 2020 to 1.4 billion by 2030, and to 2.1 billion by 2050. This demographic shift poses significant public health challenges, particularly in the management of chronic metabolic diseases. Moreover, predicting the prevalence and risk factors of NAFLD in this population is challenging due to the complexity of age-related metabolic disorders (16). Although the Rotterdam Study suggested that NAFLD prevalence stabilizes with age (17), additional studies have consistently shown that NAFLD is more prevalent in older adults compared to younger individuals (18). This discrepancy is likely because most research includes broader age ranges, whereas research specifically targeting older populations remains limited. Additionally, invasive diagnostic techniques are unsuitable for elderly individuals. Previous meta-analyses have shown that non-invasive techniques such as vibration-controlled transient elastography (VCTE), point shear wave elastography (pSWE), 2-dimensional shear wave elastography (2DSWE), and magnetic resonance elastography (MRE) (19), as well as the FibroScan-AST (FAST) score (20), may achieve diagnostic accuracy comparable to liver biopsy in diagnosing NAFLD. However, their higher medical requirements and significant cost burdens make them difficult to widely promote in most regions of the world, especially for elderly populations who cannot undergo timely monitoring at qualified hospitals on a long-term basis. Moreover, the prevalence of NAFLD is growing at a rate far exceeding previous expectations, resulting in a heavy public health burden. Therefore, it is crucial to investigate noninvasive and easily accessible indicators for predicting and diagnosing NAFLD occurrence and progression. The TyG index, by integrating triglyceride and fasting glucose levels, reflects the mechanisms of insulin resistance. It can accurately assess the degree of insulin resistance and is closely related to metabolic disruptions in pathways such as fatty acid metabolism, glucose metabolism, and insulin signaling. This study aimed to explore the diagnostic value of the TyG index, TyG-WC, TyG-BMI, and TyG-WHtR in elderly individuals with NAFLD and to examine the correlation between changes in TyG values over 2 years and the progression of hepatic steatosis. Our findings may help establish practical, non-invasive predictors for risk stratification in an aging population.

## 2 Materials and methods

### 2.1 Sources of data and samples

**Figure 1:**
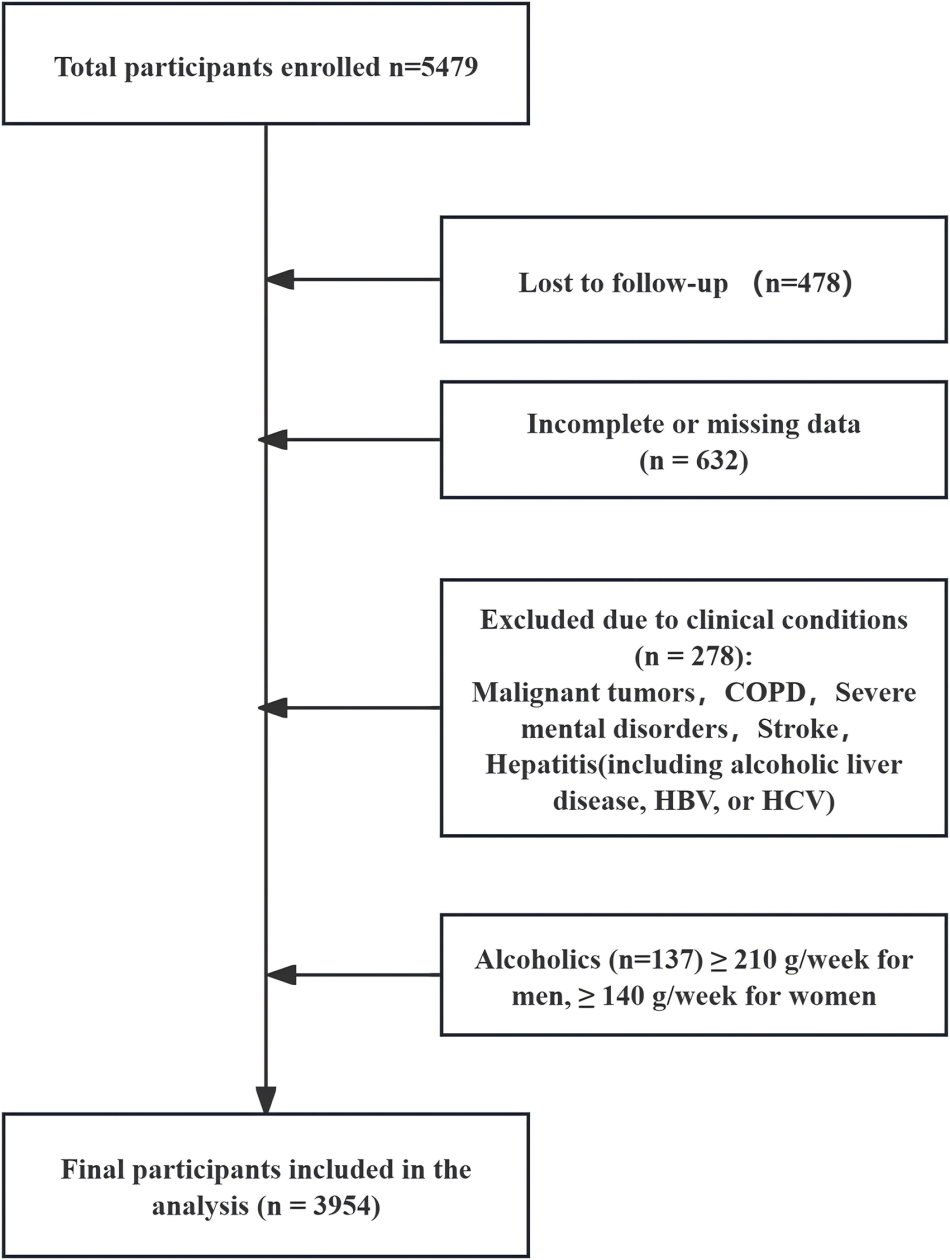
Flowchart of participant selection for the study cohort.

This study utilized physical examination data of older adults at Nan ‘an General Hospital Nan Qiao Hospital between 2022 and 2024. A retrospective cohort design was implemented with a 2-year follow-up of participants initially screened in 2022. A total of 5,479 individuals were screened, 3,954 ultimately included in the study. The exclusion criteria were as follows (Figure 1): (1) participants lost to follow-up; (2) incomplete or missing data on B-mode ultrasound or TyG index; (3) individuals with conditions that could impact the study, including malignant tumors, chronic obstructive pulmonary disease, severe mental disorders, stroke, and hepatitis (including alcoholic liver disease, hepatitis B virus [HBV], or hepatitis C virus [HCV]); and (4) individuals <60 years of age or those with excessive alcohol consumption (≥210 g/week for men and ≥140 g/week for women). This study was approved by the Ethics Committee, and written informed consent was obtained from all participants.

### 2.2 Data collection and measurement

All participants underwent a professional inquiry conducted by physicians at Hospital using a pre-designed questionnaire. Data collected included age, sex, alcohol consumption, smoking habits, exercise frequency, marital status, and medical history. Alcohol consumption was categorized based on frequency, amount per occasion, and type of alcoholic beverage, classified as never, occasional, or excessive drinking (defined as ≥210 g/week for men and ≥140 g/week for women). Participants with excessive alcohol consumption were excluded according to the study criteria.17 Smoking status was recorded as current smoker or non-smoker. Occasional exercise was defined as >90 min/week, while regular exercise was defined as > 150 min/week. Marital status was categorized as married or other (widowed, divorced, or unmarried). Height and weight were measured barefoot, using a calibrated scale. BMI was calculated as weight (kg) divided by height squared (m^2^). Waist circumference was measured using a soft tape placed horizontally around the abdomen at the level of the umbilicus during normal exhalation, ensuring the tape was snug but not compressing the tissue. The measurements were performed by experienced nurses to ensure consistency. The WHtR was calculated as waist circumference (cm) divided by height (cm). Blood samples were collected after fasting for more than 8 h and stored and transported under the same conditions using identical tubes. Whole blood cell counts were measured using a Mindray automatic hematology analyzer (BC-5180), and biochemical parameters were measured using a Hitachi automatic biochemical analyzer (7180). The change in the TyG index was assessed by comparing the measurements taken during annual health check-ups, with liver B-mode ultrasonography performed concurrently to ensure that TyG measurements corresponded with liver examination results. All anthropometric and laboratory measurements were obtained using standardized protocols. Liver ultrasonography was conducted by trained radiologists blinded to participants’ TyG index and clinical data. No study-specific interventions were administered during the 2-year follow-up period.

### 2.3 Diagnosis of NAFLD

The diagnosis of NAFLD was established based on B-mode ultrasonography findings after excluding alcohol-related liver disease, viral hepatitis (including HBV and HCV infections), drug-induced liver injury, autoimmune liver diseases, and hereditary metabolic liver diseases (21). The NAFLD diagnosis was defined by the presence of increased hepatic echogenicity (“bright liver”), attenuation of far-field echoes, and unclear visualization of intrahepatic structures (22). To further explore the different stages of fatty liver disease, B-mode ultrasonography specialists classified hepatic fat accumulation into the following categories based on the distribution of fat within the liver: (1) mild fatty liver: 5–33% hepatic fat accumulation; (2) moderate fatty liver: 34–66% hepatic fat accumulation; and (3) severe fatty liver, >66% hepatic fat accumulation (23).

### 2.4 Calculation of triglyceride-glucose (TyG) and its related indices

The TyG index was calculated as follows: TyG = ln(fasting triglycerides [mg/dL] × fasting glucose [mg/dL]/2) (24); TyG-WC = TyG × WC (25); TyG-BMI = TyG × BMI (26); and TyG-WHtR = TyG × WHtR (27).

### 2.5 Statistical analysis

All statistical analyses and visualizations were performed using IBM SPSS for Windows (version 26.0) and GraphPad (version 10.2.3). Statistical descriptions of NAFLD status were performed using independent sample t-tests and Mann–Whitney U tests. Continuous variables with normal distribution were analyzed using independent sample *t*-tests and described as mean ± SD. Non-normally distributed variables were analyzed using Mann–Whitney U tests and presented as medians and interquartile ranges (M [Q1-Q3]). For categorical variables, frequencies and chi-square tests were used to perform statistical analyses. In both univariate and multivariate analyses, binary logistic regression was employed to assess potential variables influencing NAFLD status. In the multivariable analysis, multicollinearity among independent variables was assessed using the variance inflation factor (VIF). Variables with VIF values ≥10 were considered to exhibit significant multicollinearity and were excluded from the final model. All variables retained in the final model had VIF values <10, indicating no significant multicollinearity and satisfying the model assumption. To ensure the accuracy of the model and minimize the impact of confounding factors, we included all potential confounders that could affect the results. Three models were established to evaluate the relationship between TyG index-related parameters and NAFLD. Subsequently, the samples were divided into quartiles from low to high based on their distribution, with the four groups representing the 25% lowest to highest TyG index. Binary logistic regression analysis was then performed to assess the relationship between these parameters and NAFLD. Receiver operating characteristic (ROC) curves were used to evaluate and compare the diagnostic performance of the TyG index and its related parameters across different NAFLD states. The Youden index [max (J = sensitivity + specificity − 1)] was calculated, and the corresponding parameter value was considered the optimal cutoff. Changes in NAFLD status over 2 years were categorized, and binary logistic regression was used to explore the association between changes in the TyG index and the progression of NAFLD. All statistical tests were two-tailed, and statistical significance was set at *p* < 0.05.

## 3 Results

### 3.1 Baseline characteristics of participants

This study included 3,954 individuals, with 2,087 (52.8%) in the NAFLD group and 1,887 (47.2%) in the non-NAFLD group. The average age was 72.01 ± 5.46 years. Significant differences were observed between the NAFLD and non-NAFLD groups across various parameters, including age, sex, smoking status, exercise habits, hypertension, diabetes, coronary artery disease, height, weight, BMI, waist circumference, triglyceride, uric acid, high-density lipoprotein, alanine aminotransferase (ALT), TyG, TyG-WC, TyG-BMI, and TyG-WHtR (*p* < 0.05). No statistically significant differences were found between the two groups in terms of marital status, alcohol consumption, total cholesterol, low-density lipoprotein (LDL), or aspartate aminotransferase (AST) levels (*p* > 0.05) (Table 1).

**Table 1:** Baseline clinical and biochemical characteristics of the participants.

Variables	Total (*n* = 3,954)	Non-NAFLD (*n* = 1887)	NAFLD (*n* = 2067)	*p*
Age, Mean ± SD	72.01 ± 5.46	72.61 ± 5.67	71.46 ± 5.20	**<0.001**
Sex, *n* (%)				**<0.001**
Female	2,103 (53.19)	785 (41.60)	1,318 (63.76)	
Male	1851 (46.81)	1,102 (58.40)	749 (36.24)	
Marital status, *n* (%)				0.145
Current married	3,596 (90.95)	1703 (90.25)	1893 (91.58)	
Others	358 (9.05)	184 (9.75)	174 (8.42)	
Drinking, *n* (%)				0.143
No	3,883 (98.20)	1847 (97.88)	2036 (98.50)	
Yes	71 (1.80)	40 (2.12)	31 (1.50)	
Smoking, *n* (%)				**<0.001**
No	3,128 (79.11)	1,363 (72.23)	1765 (85.39)	
Yes	826 (20.89)	524 (27.77)	302 (14.61)	
Exercise, *n* (%)				**<0.001**
Never	2,561 (64.77)	1,281 (67.89)	1,280 (61.93)	
Occasionally	1,000 (25.29)	425 (22.52)	575 (27.82)	
Frequently	393 (9.94)	181 (9.59)	212 (10.26)	
Hypertension, *n* (%)				**<0.001**
No	2,373 (60.02)	1,278 (67.73)	1,095 (52.98)	
Yes	1,581 (39.98)	609 (32.27)	972 (47.02)	
Diabetes, *n* (%)				**<0.001**
No	3,356 (84.88)	1731 (91.73)	1,625 (78.62)	
Yes	598 (15.12)	156 (8.27)	442 (21.38)	
Coronary heart disease, *n* (%)				**0.001**
No	3,881 (98.15)	1866 (98.89)	2015 (97.48)	
Yes	73 (1.85)	21 (1.11)	52 (2.52)	
Height (cm), Mean ± SD	157.14 ± 8.28	157.67 ± 8.45	156.65 ± 8.09	**<0.001**
Weight (kg), Mean ± SD	57.05 ± 9.81	53.07 ± 8.30	60.69 ± 9.68	**<0.001**
BMI (kg/m^2^), Mean ± SD	23.00 ± 3.32	21.20 ± 2.54	24.65 ± 3.08	**<0.001**
Waistline (cm), Mean ± SD	80.75 ± 9.01	76.63 ± 7.65	84.50 ± 8.51	**<0.001**
Glucose (mmol/L), Mean ± SD	5.76 ± 1.96	5.38 ± 1.60	6.10 ± 2.18	**<0.001**
TG (mmol/L), Mean ± SD	1.59 ± 1.12	1.24 ± 0.62	1.91 ± 1.36	**<0.001**
UA (μmol/L), Mean ± SD	369.15 ± 95.95	355.25 ± 91.55	381.83 ± 98.12	**<0.001**
TC (mmol/L), Mean ± SD	5.43 ± 1.09	5.42 ± 1.03	5.45 ± 1.14	0.49
LDL (mmol/L), Mean ± SD	3.27 ± 0.89	3.25 ± 0.85	3.30 ± 0.92	0.058
HDL (mmol/L) Mean ± SD	1.68 ± 0.37	1.78 ± 0.38	1.58 ± 0.34	**<0.001**
ALT (U/L), M (Q_1_, Q_3_)	16.70 (13.20, 21.80)	15.10 (12.20, 19.40)	18.40 (14.50, 24.30)	**<0.001**
AST (U/L), M (Q_1_, Q_3_)	20.00 (17.00, 24.00)	20.20 (17.10, 24.00)	19.80 (16.90, 23.90)	0.24
TyG quantile, *n* (%)				**<0.001**
<8.29	988 (24.99)	746 (39.53)	242 (11.71)	
≥8.29, <8.64	989 (25.01)	556 (29.46)	433 (20.95)	
≥8.64, < 9.04	987 (24.96)	381 (20.19)	606 (29.32)	
≥9.04	990 (25.04)	204 (10.81)	786 (38.03)	
TyG-WC quantile, *n* (%)				**<0.001**
<632.30	989 (25.01)	812 (43.03)	177 (8.56)	
≥632.30, <696.92	988 (24.99)	597 (31.64)	391 (18.92)	
≥696.92, <769.86	988 (24.99)	352 (18.65)	636 (30.77)	
≥769.86	989 (25.01)	126 (6.68)	863 (41.75)	
TyG-BMI quantile, *n* (%)				**<0.001**
<174.47	989 (25.01)	847 (44.89)	142 (6.87)	
≥174.47, < 197.77	988 (24.99)	626 (33.17)	362 (17.51)	
≥197.77, <224.55	988 (24.99)	315 (16.69)	673 (32.56)	
≥224.55	989 (25.01)	99 (5.25)	890 (43.06)	
TyG-WHtR quantile, *n* (%)				**<0.001**
<4.00	989 (25.01)	834 (44.20)	155 (7.50)	
≥4.00, <4.45	988 (24.99)	587 (31.11)	401 (19.40)	
≥4.45, <4.93	988 (24.99)	322 (17.06)	666 (32.22)	
≥4.93	989 (25.01)	144 (7.63)	845 (40.88)	

*t*, *t*-test; Z, Mann–Whitney test; *χ*^2^, Chi-square test. SD, standard deviation; M, Median; Q_1_, 1st Quartile; Q_3_, 3st Quartile. TG, triglycerides; TC, total cholesterol; UA, uric acid; LDL, low-density lipoprotein cholesterol, HDL, high-density lipoproteincholesterol; ALT, alanine aminotransferase; AST, aspartate aminotransferase. Bold values indicate statistical significance at *p* < 0.05.

### 3.2 Relationship between the TyG index and its derivatives with NAFLD

We divided the TyG index and its related parameters into quartiles for comparison, and significant statistical differences were observed among the quartiles for all four parameters (*p* < 0.001).

Univariate analysis revealed that age, sex, smoking status, hypertension, diabetes, coronary heart disease, height, weight, BMI, waist circumference, triglycerides, uric acid, high-density lipoprotein (HDL), ALT, TyG, TyG-WC, TyG-BMI, and TyG-WHtR were significantly associated with the incidence of NAFLD (*p* < 0.05) (Table 2). In the multivariate analysis of these baseline data, we employed the VIF to avoid multicollinearity and excluded total cholesterol, LDL, weight, waist circumference, and WHtR (VIF ≥ 10). After adjusting for potential confounders, we established three models to explore the impact of different factors on TyG, TyG-WC, TyG-BMI, and TyG-WHtR.

**Table 2:** Multivariate analysis results.

Model 1		
Variables	OR (95% CI)	*p* value
TyG		
<8.29	1.00 (Reference)	
≥8.29, <8.64	2.33 (1.92 ~ 2.83)	<0.001
≥8.64, <9.04	4.61 (3.79 ~ 5.62)	<0.001
≥9.04	10.91 (8.80 ~ 13.53)	<0.001
TyG-WC		
<632.30	1.00 (Reference)	
≥632.30, <696.92	3.20 (2.59 ~ 3.97)	<0.001
≥696.92, <769.86	9.30 (7.48 ~ 11.56)	<0.001
≥769.86	37.49 (28.94 ~ 48.56)	<0.001
TyG-BMI		
<174.47	1.00 (Reference)	
≥174.47, <197.77	3.34 (2.68 ~ 4.18)	<0.001
≥197.77, <224.55	12.41 (9.90 ~ 15.55)	<0.001
≥224.55	49.84 (37.82 ~ 65.69)	<0.001
TyG-WHtR		
<4.00	1.00 (Reference)	
≥4.00, <4.45	3.64 (2.93 ~ 4.52)	<0.001
≥4.45, <4.93	11.07 (8.87 ~ 13.83)	<0.001
≥4.93	29.48 (22.91 ~ 37.94)	<0.001

Model 2		
Variables	OR (95% CI)	*p* value
TyG		
<8.29	1.00 (Reference)	
≥8.29, <8.64	1.90 (1.55 ~ 2.34)	<0.001
≥8.64, <9.04	2.92 (2.36 ~ 3.62)	<0.001
≥9.04	4.59 (3.58 ~ 5.89)	<0.001
TyG-WC		
<632.30	1.00 (Reference)	
≥632.30, <696.92	2.69 (2.15 ~ 3.35)	<0.001
≥696.92, <769.86	6.47 (5.13 ~ 8.15)	<0.001
≥769.86	20.64 (15.62 ~ 27.28)	<0.001
TyG-BMI		
<174.47	1.00 (Reference)	
≥174.47, <197.77	2.75 (2.18 ~ 3.46)	<0.001
≥197.77, <224.55	8.58 (6.73 ~ 10.94)	<0.001
≥224.55	29.40 (21.76 ~ 39.74)	<0.001
TyG-WHtR		
<4.00	1.00 (Reference)	
≥4.00, <4.45	2.78 (2.22 ~ 3.48)	<0.001
≥4.45, <4.93	7.05 (5.57 ~ 8.94)	<0.001
≥4.93	15.09 (11.49 ~ 19.82)	<0.001

Model 3		
Variables	OR (95% CI)	*p* value
TyG		
<8.29	1.00 (Reference)	
≥8.29, <8.64	1.89 (1.53 ~ 2.32)	<0.001
≥8.64, <9.04	2.84 (2.29 ~ 3.52)	<0.001
≥9.04	4.36 (3.39 ~ 5.61)	<0.001
TyG-WC		
<632.30	1.00 (Reference)	
≥632.30, <696.92	2.64 (2.12 ~ 3.30)	<0.001
≥696.92, <769.86	6.32 (5.01 ~ 7.97)	<0.001
≥769.86	19.77 (14.92 ~ 26.22)	<0.001
TyG-BMI		
<174.47	1.00 (Reference)	
≥174.47, <197.77	2.73 (2.17 ~ 3.44)	<0.001
≥197.77, <224.55	8.43 (6.60 ~ 10.76)	<0.001
≥224.55	28.23 (20.81 ~ 38.28)	<0.001
TyG-WHtR		
<4.00	1.00 (Reference)	
≥4.00, <4.45	2.75 (2.19 ~ 3.45)	<0.001
≥4.45, <4.93	6.93 (5.46 ~ 8.79)	<0.001
≥4.93	14.56 (11.05 ~ 19.19)	<0.001

Model 1: Adjusted for age and sex. Model 2: Adjusted for age, sex, uric acid, ALT, AST, smoking status, and HDL. Model 3: Further adjusted for marital status, alcohol consumption, smoking status, exercise, hypertension, diabetes, coronary heart disease, uric acid, HDL, ALT, and AST.

Model 1, which minimally adjusted for age and sex, showed that the TyG-BMI group had the highest odds ratio (OR). Using the first quartile (Q1) as the reference, the OR for the fourth quartile (Q4) was 49.84 (95% CI, 37.82 ~ 65.69) (*p* < 0.001). In Model 2, after adjusting for sex, age, uric acid, ALT, AST, smoking status, and HDL, TyG-BMI remained the most prominent indicator, with an OR of 29.40 (95% CI, 21.76 ~ 39.74) (*p* < 0.001) when comparing Q4 to Q1. In Model 3, after further adjusting for age, sex, marital status, alcohol consumption, smoking status, exercise, hypertension, diabetes, coronary heart disease, uric acid, HDL, ALT, and AST, the OR for TyG-BMI Q4 was 28.23 (95% CI, 20.81 ~ 38.28) (*p* < 0.001) (Table 2).

From the results of the three models, it can be concluded that higher TyG, TyG-WC, TyG-BMI, and TyG-WHtR levels were significant risk factors for NAFLD. The top three risk factors by OR ranking were TyG-BMI, TyG-WC, and TyG-WHtR.

### 3.3 Predictive value of each parameter

**Figure 2:**
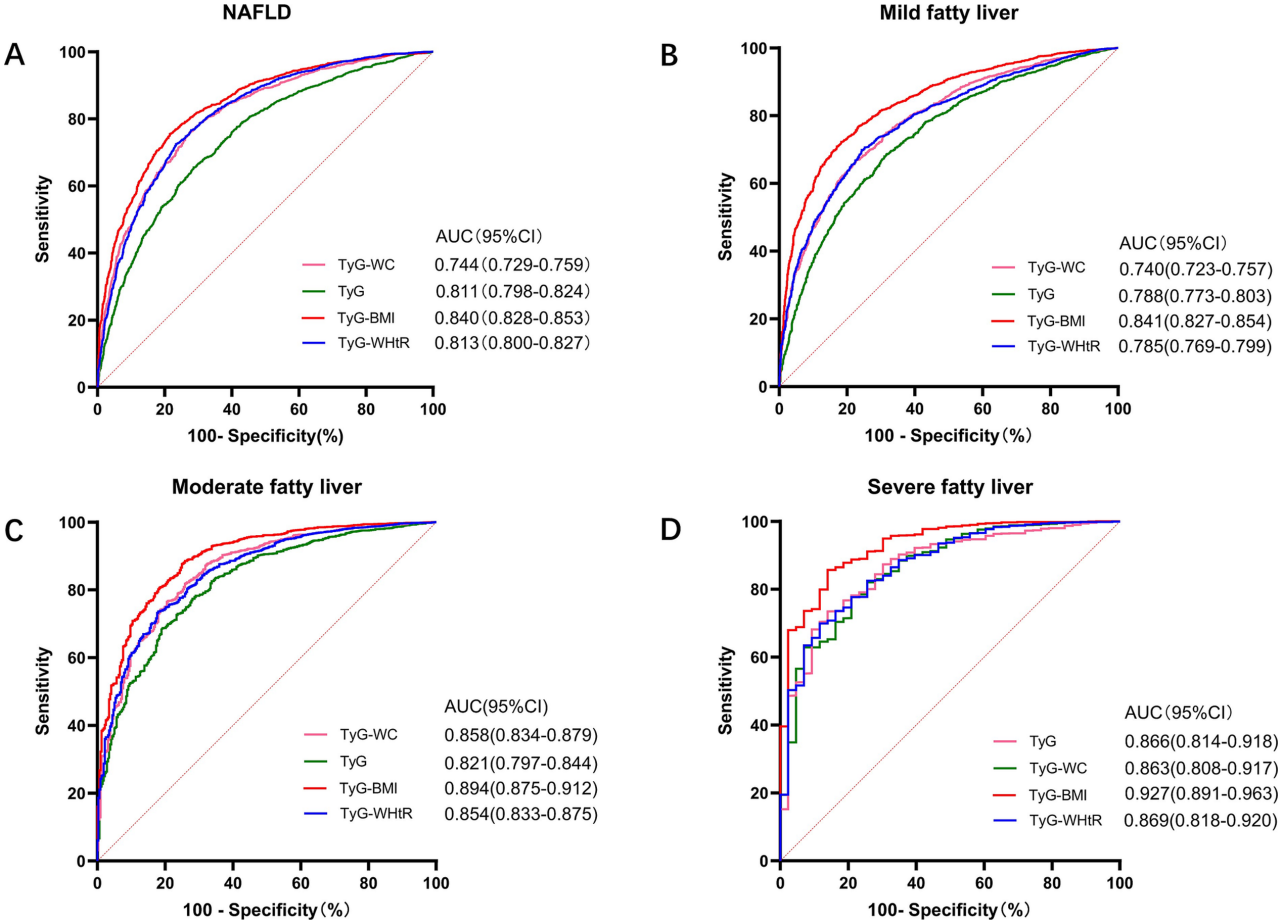
Receiver operating characteristic (ROC) curves and area under the curve (AUC) values for the four parameters (TyG index, TyG-WC, TyG-BMI, and TyG-WHtR) were used to evaluate their diagnostic performance in detecting NAFLD, mild, moderate, and severe hepatic steatosis. **(A)** Diagnostic ability of the four parameters for identifying NAFLD was assessed. **(B)** Ability of the four parameters to detect mild hepatic steatosis was evaluated. **(C)** Performance of the four parameters in identifying moderate hepatic steatosis was analyzed. **(D)** Predictive power of the four parameters in detecting severe hepatic steatosis was examined.

To further investigate the impact of these four parameters on NAFLD, we generated ROC curves to assess their predictive values. In the general population, all four parameters significantly predicted NAFLD status (*p* < 0.001), with area under the curve (AUC) values ranging from 0.744 to 0.840. Within the NAFLD population, the disease was categorized into mild, moderate, and severe stages based on B-mode ultrasound findings. We evaluated the ability of these four parameters to predict each NAFLD stage. The results indicated that all four parameters exhibited significant predictive power across the three ROC curves. The top three predictors were TyG-BMI, TyG-WHtR, and TyG-WC. In the overall population, the AUCs (95% CI) for predicting NAFLD were 0.840 (0.828–0.853), 0.813 (0.800–0.827), and 0.811 (0.798–0.824), respectively. In the ROC curve for severe NAFLD, these four parameters demonstrated the strongest predictive power, with AUCs (95% CI) of 0.927 (0.891–0.963), 0.869 (0.818–0.920), and 0.863 (0.808–0.917) (Figure 2). Based on the ROC curve and Youden index, the optimal cutoff value for TyG-BMI in predicting NAFLD was 197.87, with a sensitivity of 75.6% and a specificity of 78.4%. The sensitivities for predicting mild, moderate, and severe NAFLD were 83, 74.1, and 86%, respectively, and the specificities were 62.9, 73.1, and 66.4%, respectively.

### 3.4 Relationship between TyG index changes and the progression of hepatic steatosis from 2022 to 2024

**Figure 3:**
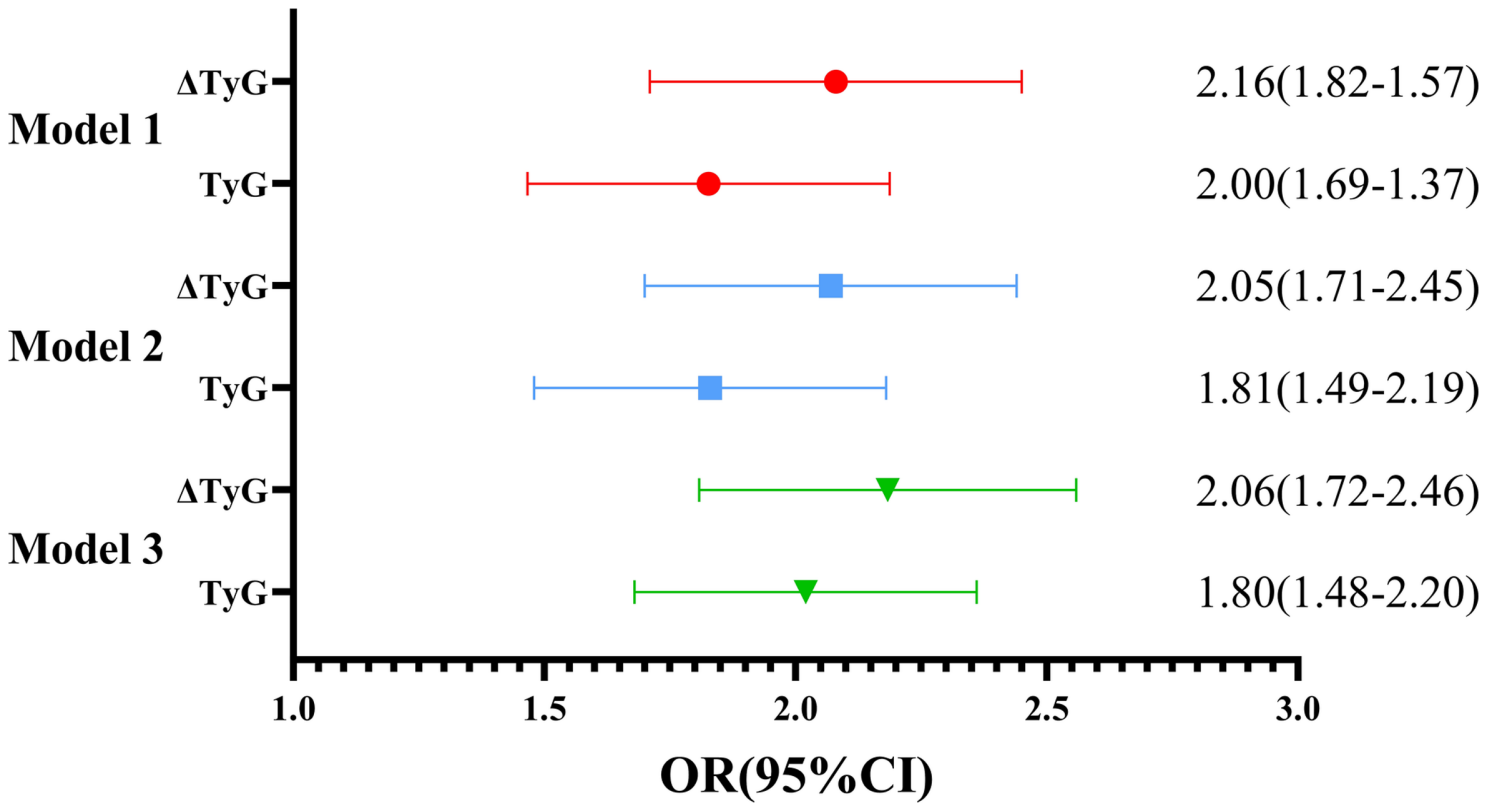
Comparison of the impact of TYG baseline values and two-year dynamic changes on outcomes.

Liver ultrasonography was repeated in the same cohort in 2024 and compared with baseline assessments from 2022. Based on changes in hepatic steatosis severity, participants were classified into a progression group (aggravated) and a stable/improvement group. Notably, in all three models, ΔTyG showed a stronger association with disease progression compared to the baseline TyG index, indicating its superior predictive value. Specifically, the ORs for baseline TyG ranged from 1.80 to 2.00 across the models while the ORs for ΔTyG were consistently higher, ranging from2.06 to 2.16. After full adjustment for potential confounders, the association between ΔTyG and NAFLD progression remained consistently stronger and more stable compared to baseline TyG, suggesting that longitudinal changes in TyG may provide a more robust indicator of disease dynamics. Detailed results are presented in Figure 3.

## 4 Discussion

In this study, we reached the following conclusions: (1) The TyG index and its related indices were highly correlated with NAFLD across different models. (2) The TyG index and its related indices demonstrated significant predictive value for both NAFLD and its varying degrees of severity. (3) Monitoring changes in the TyG index can provide insights into the progression or regression trends of NAFLD. The following sections present a detailed discussion of these findings.

Recent studies have demonstrated a strong correlation between the TyG index and related indices in NAFLD. Peng et al. suggested through their research that the TyG index is a crucial indicator of the risk of NAFLD (13). In a study involving 10,390 participants, the clinical value of the TyG index in predicting the risk of all-cause mortality in individuals with NAFLD was analyzed and confirmed. Furthermore, they highlighted its importance as a key indicator for monitoring the progression of NAFLD to liver fibrosis and underscored its role in follow-up and preventive strategies (27). A study involving 1,727 adults demonstrated that the TyG index and its related parameters can be effectively utilized for early screening and monitoring of disease progression in individuals with NAFLD (14). These studies collectively confirm the significance of the TyG index and related indices in identifying NAFLD pathology. Moreover, they demonstrated an independent positive association between TyG-BMI and the risk of NAFLD, offering new insights into the prevention and treatment of the disease (28). In this study, we aimed to explore the relationship between the TyG index and its related indices and the severity of NAFLD in an elderly population. Additionally, we sought to determine whether monitoring the changes in the TyG index could accurately assess NAFLD progression.

Fatty liver development is thought to result from the breakdown of triglycerides in adipose tissue into fatty acids, which are then transported to the liver through the bloodstream. This process is regulated by insulin on adipocytes. When insulin receptor signaling in adipose tissue is impaired, leading to insulin resistance, the balance of lipolysis is disrupted. Under these conditions, an excessive amount of fatty acids is transported to the liver (29). Insulin resistance (IR) is the primary cause of this imbalance (30). Since Simental-Mendia et al. (31) first introduced the concept of calculating the TyG index using fasting glucose and triglyceride levels, it has demonstrated significant efficacy in assessing insulin resistance, cardiovascular disease, all-cause mortality, and NAFLD (32). Emerging evidence suggests that NAFLD may be a poor prognostic factor for bladder cancer(BC), and the TyG index has shown high sensitivity in identifying individuals at risk of BC (33, 34). These findings highlight the broader clinical utility of the TyG index beyond liver-related outcomes. An increase in the TyG index often indicates impaired pancreatic *β*-cell function (35), reflecting not only the state of IR but also its close association with the onset and progression of various diseases (36). This, in turn, influences the development of NAFLD, potentially explaining the strong association observed between the TyG index and the occurrence and progression of NAFLD. For the elderly, this situation may be even more complex. Due to physiological decline and intricate immune responses, there may be additional factors influencing the development of fatty liver (37). For example, a significant decrease in mitochondrial oxidation and phosphorylation activity has led to enhanced insulin resistance in the elderly population, increasing their likelihood of developing NAFLD (38). Therefore, under such a complex set of risk factors, we aimed to investigate whether the correlation between the TyG index and NAFLD remains consistent with that reported in previous studies. Additionally, obesity indices, such as BMI, WC, and WHtR, are also important indicators for assessing NAFLD (11, 12). Therefore, combining TyG and obesity indices to derive a new parameter may enable a more accurate and sensitive assessment of NAFLD and its various stages. In our study, we simultaneously incorporated four parameters to summarize and compare the risk factors associated with NAFLD and predictive states of the disease.

Previous studies generally regarded middle-aged and elderly individuals as having a high prevalence of NAFLD (39). Based on these findings, age may influence the diagnostic value of the TyG index for fatty liver disease (9). However, research on the diagnostic value of TyG and its related indices in elderly populations remains limited. We investigated the role of TyG in NAFLD in 3,954 older adults. In this study, we established three models to confirm whether TyG levels and related indices are independent risk factors for NAFLD. Our findings indicate that even in the complex immunological landscape of the elderly, indices such as TyG can accurately reflect their correlation with NAFLD, providing evidence to support the clinical diagnosis of NAFLD in this age group. ROC curve analysis revealed that TyG and its related indices possess significant predictive value for different stages of NAFLD, particularly TyG-BMI, which demonstrated high predictive value across all three disease stages. This suggests that these indices may facilitate a more accurate and sensitive assessment of NAFLD status and severity in clinical practice. Although imaging techniques are widely used as the primary diagnostic approach for NAFLD worldwide (40), their accuracy can be compromised by confounding factors, such as chronic liver disease and liver fibrosis, which may interfere with the specific evaluation of disease status (41). The TyG index and related indices, when combined with imaging assessments, provide clinicians with valuable insights into the diagnosis and management of various diseases. Moreover, the predictive efficacy demonstrated by these four parameters can significantly enhance diagnostic capabilities. Numerous studies have confirmed that the TyG index can predict the prevalence of NAFLD; however, most of these studies were cross-sectional in nature, and the impact of changes in the TyG index on the varying outcomes of NAFLD is yet to be established. A meta-analysis of randomized controlled trials indicated that approximately 20% of patients with NAFLD may rapidly progress to advanced cirrhosis within a short period without exhibiting any clinical symptoms (42), However, the methods for identifying such rapid progression remain unclear. Therefore, we conducted a two-year follow-up study in this population to determine whether changes in the TyG index affect the progression of NAFLD. Over time, the progression of NAFLD results in various outcomes (43). These results indicate that as the TyG index increases, there is a progressive worsening trend in the severity of NAFLD, suggesting that monitoring changes in the TyG index can be a useful tool for assessing disease progression. This trend was particularly pronounced in groups with prior worsening and improvement of NAFLD, where an increase in the TyG index serves as a valuable risk indicator for predicting changes. In the future, combining TyG index with B-mode ultrasonography may help screen for high-risk populations with a potential progression of NAFLD, providing clinicians with more accurate non-invasive diagnostic tools, such as the FibroScan-AST (FAST) score and transient elastography. This approach could not only effectively reduce the waste of medical resources but also support early intervention, thereby improving patient outcomes.

With the increasing prevalence of NAFLD, it has become a significant cause of hepatocellular carcinoma (HCC) (44); however, there are currently no specific medications available for the treatment of NAFLD in clinical practice. Instead, management strategies primarily involve interventions such as dietary modifications and weight control to mitigate the effects of NAFLD (45). This treatment approach may be effective for early-stage NAFLD; however, as the progression of NAFLD advances, relying merely on dietary changes and weight management is insufficient to halt disease progression. There is a pressing need to implement additional therapeutic measures, including pharmacological interventions, aimed at controlling blood glucose levels and reducing blood lipids (46). The TyG index was calculated based on blood glucose and lipid levels. Monitoring changes in the TyG index not only allows for the prediction of the severity of NAFLD but also provides a dynamic assessment of disease progression. Moreover, compared with other indices, the TyG index and its related parameters demonstrate a significantly higher sensitivity in predictive value (13), which may effectively assist clinicians in implementing interventions such as blood glucose control and lipid-lowering medications when a sustained increase in the TyG index is observed, thereby preventing further progression of NAFLD. Although the effectiveness of the TyG index has been supported by numerous studies, factors such as regional differences in diet and lifestyle may influence its performance. Therefore, future research should focus on global validation and the establishment of standardized protocols to ensure the TyG index’s applicability and accuracy across different regions.

Our study has some limitations. Firstly, it diagnosed and classified NAFLD solely using B-mode ultrasound, which prevented the determination of whether patients would progress to NASH. Although simple steatosis is generally accepted as a diagnostic criterion for NAFLD, the limitations of this method must be considered. Secondly, owing to technical and financial constraints, we were unable to assess the degree of liver fibrosis using a liver biopsy. Several potential confounding factors may have influenced the observed association between changes in the TyG index and the progression of NAFLD/MASLD. Lifestyle modifications during the follow-up period, such as changes in dietary intake, physical activity, or body weight, could affect insulin resistance and lipid metabolism, thereby impacting TyG levels (47). In addition, comorbidities including diabetes, hypertension, and dyslipidemia (48), as well as the initiation or adjustment of relevant medications (e.g., statins, antihyperglycemic agents) (49), may have influenced both TyG dynamics and hepatic fat accumulation. Although our multivariable models adjusted for many of these covariates, residual confounding cannot be entirely ruled out due to the lack of detailed longitudinal data on lifestyle and pharmacologic interventions.

Despite these limitations, our findings underscore the clinical utility of TyG-based indices as practical and noninvasive tools for the screening and monitoring of NAFLD/MASLD. The TyG index, calculated from routine fasting glucose and triglyceride measurements, serves as a surrogate marker for insulin resistance and can be readily integrated into standard clinical workflows. Importantly, our results indicate that not only baseline TyG levels but also longitudinal increases in TyG are significantly associated with disease progression. This highlights the value of repeated TyG assessments over time to identify individuals at elevated risk, especially in older populations where scalable, cost-effective screening strategies are particularly needed.

## 5 Conclusion

In elderly populations, the TyG index and its related parameters hold significant value in the diagnosis of NAFLD and prediction of different stages of the disease. Additionally, monitoring changes in the TyG index can effectively indicate NAFLD progression. Given its simplicity and availability in routine laboratory testing, the TyG index may serve as a practical tool for early detection and longitudinal monitoring of NAFLD/MASLD in primary care settings. Incorporating TyG-based metrics into community health screening programs or clinical guidelines may facilitate timely intervention and help mitigate long-term hepatic and metabolic complications, particularly in aging populations.

## Data Availability

The raw data supporting the conclusions of this article will be made available by the authors, without undue reservation.
