# Correction: Triglyceride-glucose index and related parameters in the diagnosis and predictive efficacy for older adults with NAFLD/MASLD: a prospective cohort study

**DOI:** 10.3389/fmed.2026.1804927

**Published:** 2026-03-18

**Authors:** Yuwei Chen, Xinjun Lin, Yanqiu Chen, Bingcai Wang, Chaoxiang Xu, Yaoguo Wang

**Affiliations:** 1 Department of Cardiology, The Second Affiliated Hospital of Fujian Medical University, Quanzhou, Fujian, China; 2 Department of Ultrasound Medicine, The Second Affiliated Hospital of Fujian Medical University, Quanzhou, Fujian, China

**Keywords:** triglyceride-glucose index, non-alcoholic fatty liver disease, triglyceride-glucose waistline, triglyceride-glucose BMI, triglyceride-glucose waist-to-height ratio, Fujian Province, China

The funder Quanzhou Science and Technology Bureau, project funding No. 2023NS001 was erroneously omitted. The correct funding statement is:

“The author(s) declare that financial support was received for the research and/or publication of this article. This study was supported by Quanzhou Science and Technology Bureau, No. 2023NS001.”

The original version of this article has been updated.

